# The Use of Heated Tobacco Products is Associated with Asthma, Allergic Rhinitis, and Atopic Dermatitis in Korean Adolescents

**DOI:** 10.1038/s41598-019-54102-4

**Published:** 2019-11-27

**Authors:** Ahnna Lee, Sook Young Lee, Kang-Sook Lee

**Affiliations:** 1 Department of Public Health, Graduate School, The Catholic University of Korea, Seoul, Korea; 2 Department of Preventive Medicine, College of Medicine, The Catholic University of Korea, Seoul, Korea; 3 Division of Pulmonary, Allergy and Critical Care Medicine, Department of Internal Medicine, College of Medicine, Seoul St. Mary’s Hospital, The Catholic University of Korea, Seoul, Korea

**Keywords:** Asthma, Atopic dermatitis, Epidemiology, Risk factors, Allergy

## Abstract

The increasing use of new and emerging tobacco products has raised public health concern worldwide. This study aimed to assess the association between tobacco product use and the risk of allergic diseases. We used cross-sectional data of 58,336 students aged 12–18 years from the 2018 Korea Youth Risk Behavior Survey. This study considered three tobacco products, namely cigarettes, electronic cigarettes (e-cigarettes), and heated tobacco products. Descriptive analyses, as well as simple and multinomial logistic regression analyses with a complex sampling design, were performed. Multiple tobacco use had an association with the risk of each allergic disease. Use of each tobacco product was significantly associated with an increased risk of multi-morbidity of asthma, allergic rhinitis, and atopic dermatitis. Furthermore, lifetime use of each tobacco product was associated with the prevalence of atopic dermatitis. This highlights the importance of paying close attention to smoking by adolescents and its association with allergy epidemics. Future research should consider intensity of smoking and/or severity of allergic symptoms.

## Introduction

Allergic diseases have been considered as a serious public health concern, as they place significant economic burden on health services and lead to diminished quality of life^1^. Allergic diseases, such as asthma, allergic rhinitis (AR), and atopic dermatitis (AD), are often associated with sleep disturbance, intellectual dysfunction, and activity restriction, leading to physical and mental suffering^2^. In addition, the onset of such allergic diseases in childhood or adolescence may persist into adulthood^3,4^, requiring an integrated approach to proper treatment and management.

During the past decade (2009–2018), the prevalence of asthma, AR, and AD among high school students in Korea increased from 7.4% to 8.9%, 29.6% to 38.7%, and 18.2% to 26.1%, respectively^5^. The socioeconomic costs of allergic diseases in Korea were calculated to be approximately $1.8 billion, equivalent to 0.15% of the nation’s total gross domestic product of $1.3 trillion. In 2014, the socioeconomic costs of AR reached $1.1 billion (58.6%), while those of asthma and AD amounted to $0.62 billion (33.2%) and $0.16 billion (8.3%)^6^.

Tobacco smoke exposure, either active or passive, has been identified as a factor that increases the prevalence of asthma, AR, and AD^7–9^. Numerous toxic chemical and carcinogens, which contribute to respiratory irritation, such as acrolein, acetaldehyde, and formaldehyde, have been found in cigarette smoke^10^. According to laboratory studies with animal models^11,12^ and a literature review study^13^, tobacco smoke could affect inflammatory processes, leading to an increase in eosinophils, collagen production, and airway remodeling.

Unlike combusted cigarettes, electronic cigarettes (e-cigarettes) heat a metal coil to aerosolize a liquid mixture of nicotine, glycerol, in addition to flavoring agents^14^. The vapor produced by e-cigarettes has been reported to induce oxidative stress and suppress immune-related gene expression, leading to toxicity and impaired host defense mechanisms^15–17^. A plethora of research^18^ has been conducted on the association of tobacco smoke exposure with asthma, AR, and AD, whereas only a few studies focusing on the association between e-cigarettes and allergic diseases have been published.

Heated tobacco products (HTPs), the latest type of tobacco product, heat a processed tobacco leaf, not a liquid, up to 350 °C^19^. According to certain studies^20,21^, HTPs have the same harmful constituents as conventional cigarettes. Additionally, recent findings^22^ show that they can potentially cause impairments in lung cells by altering mitochondrial functionality, inducing oxidative stress, and limiting airflow. These impairments are often associated with asthma and chronic obstructive pulmonary disease. A recent *in vitro* study^23^ confirmed that HTP aerosols had adverse effects on human bronchial epithelial cells and that their cytotoxicity was higher than that of e-cigarettes, but less than that of conventional cigarettes.

This study aimed to assess the impact of tobacco use, including HTPs, on asthma, AR, and AD in Korean adolescents. The use of multiple tobacco products and comorbidity of allergic diseases were also considered in this study. To the best of our knowledge, this is the very first study to evaluate the effects of tobacco product use on asthma, AR, and AD with respect to multiple tobacco product use and development of multiple morbidities.

## Results

Of all participants, 2.4% (1,443/58,336), 20.9% (11,884/58,336), and 7.2% (4,198/58,336) reported the diagnosis of asthma, AR, and AD within the past year, respectively (Table 1). In total, 25.8% of adolescents were affected by any of the three allergic diseases. Among the three allergic diseases, participants with AR had the oldest mean age of onset (15.2 years).

**Table 1:** General characteristics of participants.

Characteristics		Total Participants	Normal	Asthma	*p*-value	Allergic Rhinitis	*p*-value	Atopic Dermatitis	*p*-value
Total		58,336	43,507 (74.2)	1443 (2.4)		11,884 (20.9)		4198 (7.2)	
Age^†^		15.0 (1.8)	14.7 (1.8)	14.9 (1.7)	0.006*	15.2 (1.8)	<0.001*	15.1 (1.7)	0.016*
Sex	Male	29,613	22,526 (53.2)	853 (2.8)	<0.001*	5746 (19.8)	<0.001*	1770 (6.0)	<0.001*
	Female	28,723	20,981 (46.8)	590 (2.1)		6138 (22.0)		2428 (8.5)	
Obesity	Underweight	2916	2238 (4.9)	64 (2.1)	0.009*	547 (19.9)	0.450*	181 (6.4)	0.005*
	Normal or Healthy Weight	46,670	34,830 (80.1)	1127 (2.4)		9543 (20.9)		3306 (7.1)	
	Overweight	5834	4283 (9.8)	175 (3.1)		1205 (21.3)		473 (8.1)	
	Obese	2916	2156 (5.1)	77 (2.6)		589 (20.3)		237 (8.0)	
Residential area	Large city	25,951	19,340 (43.1)	649 (2.5)	0.409	5349 (20.8)	<0.001*	1802 (6.9)	0.047*
	Medium, small city	28,030	20,684 (50.5)	697 (2.4)		5896 (21.6)		2090 (7.4)	
	Province	4355	3483 (6.4)	97 (2.1)		639 (15.1)		306 (7.4)	
Family economic status	High	23,603	17,343 (40.4)	600 (2.5)	0.004*	5108 (22.1)	<0.001*	1632 (6.9)	<0.001*
	Middle	27,139	20,597 (47.0)	618 (2.3)		5192 (19.6)		1931 (7.1)	
	Low	7594	5567 (12.6)	225 (2.9)		1584 (21.2)		635 (8.5)	
Physical activity	<5 days/week	50,011	37,349 (86.2)	1192 (2.4)	0.003*	10,135 (20.7)	0.112	3618 (7.2)	0.188
	≥5 days/week	8325	6158 (13.8)	251 (2.9)		1749 (21.5)		580 (6.8)	

^*^Significance at *p* < 0.05.

^†^Age, year (SD).

Conventional cigarettes, e-cigarettes, and HTPs had been used by 13.9% (8,129/58,336), 7.1% (4,114/58,336) and 2.4% (1,414/58,336) of participants (Table 2). The allergic disease group used conventional cigarettes, e-cigarettes, and HTPs more than the group without allergic diseases. Secondhand smoking at home and at school was more prevalent in the asthma, AR, and AD groups.

**Table 2:** Associations between smoking-related characteristics and the presence of Asthma, Allergic Rhinitis, and Atopic Dermatitis within the past 12 months.

Smoking-related characteristics		Total Participants	Normal	Asthma	*p*-value	Allergic Rhinitis	*p*-value	Atopic Dermatitis	*p*-value
Ever use of cigarettes	Yes	8129	5948 (14.2)	257 (3.2)	<0.001*	1729 (22.0)	0.009*	641 (8.1)	<0.001*
	No	50,207	37,559 (85.8)	1186 (2.3)		10,155 (20.7)		3557 (7.0)	
Ever use of e-cigarettes^†^	Yes	4114	2986 (7.4)	128 (3.1)	0.007*	895 (22.2)	0.031*	347 (8.3)	0.003*
	No	54,222	40,521 (92.6)	1315 (2.4)		10,989 (20.7)		3851 (7.1)	
Ever use of HTP^†^	Yes	1414	962 (2.4)	64 (4.3)	<0.001*	347 (24.4)	0.002*	142 (9.9)	<0.001*
	No	56,922	42,545 (97.6)	1379 (2.4)		11,537 (20.8)		4056 (7.1)	
Secondhand smoking at home	0 day/week	44,907	33,709 (78.0)	1056 (2.3)	<0.001*	9016 (20.5)	0.001*	3068 (6.8)	<0.001*
	1–4 days/week	9724	7148 (16.0)	253 (2.5)		2045 (21.6)		772 (8.0)	
	≥5 days/week	3705	2650 (6.0)	134 (3.7)		823 (22.8)		358 (9.9)	
Secondhand smoking at school	0 day/week	47,293	35,267 (81.2)	1081 (2.2)	<0.001*	9355 (20.2)	<0.001*	3288 (6.9)	<0.001*
	1–4 days/week	8540	6109 (14.4)	272 (3.1)		1962 (23.5)		675 (7.8)	
	≥5 days/week	2503	1771 (4.3)	90 (3.7)		567 (23.0)		235 (9.4)	
Tobacco use type	Ever use	8794 (100)							
	Cigarettes only	4496 (49.1)	3337 (7.7)	137 (3.0)	<0.001*	917 (20.9)	0.004*	333 (7.8)	<0.001*
	E-cigarettes only	540 (6.1)	413 (1.0)	18 (3.4)		96 (17.1)		50 (8.2)	
	HTP only	51 (0.6)	32 (0.1)	5 (8.1)		15 (27.8)		4 (7.7)	
	Cigarettes + E-cigarettes	2344 (27.6)	1733 (4.3)	63 (2.8)		497 (22.3)		175 (7.7)	
	Cigarettes + HTP	133 (1.5)	90 (0.2)	12 (9.6)		30 (25.4)		16 (14.1)	
	E-cigarettes + HTP	74 (0.9)	52 (0.1)	2 (2.7)		17 (19.1)		5 (6.4)	
	Cigarettes + E-cigarettes + HTP	1156 (13.8)	788 (2.0)	45 (3.7)		285 (24.5)		117 (9.7)	
	Never use	49,542	37,062 (84.6)	1161 (2.3)		10,027 (20.7)		3498 (7.0)	

^*^Significance at *p* < 0.05. ^†^Electronic cigarettes: E-cigarettes, HTP: Heated Tobacco Product.

Table 3 shows that the three allergic diseases were more likely to be found among participants reporting that they had ever used conventional cigarettes or HTPs. Having asthma, AR, and AD was positively and significantly associated with secondhand smoking at home and school for five or more days per week. Regarding multiple tobacco product use, 57.1% of e-cigarette users were dual users of conventional cigarettes and e-cigarettes. Interestingly, 82.4% of HTP users were triple users of cigarettes, e-cigarettes, and HTPs, whereas 28.6% and 15.0% of ever e-cigarette and conventional cigarette users were triple users, respectively.

**Table 3:** Multiple logistic regression model of the association of smoking-related characteristics with Asthma, Allergic Rhinitis, and Atopic Dermatitis.

Smoking-related characteristics		Asthma		Allergic Rhinitis		Atopic Dermatitis	
		OR (95% CI)		OR (95% CI)		OR (95% CI)	
		Unadjusted	Adjusted^‡^	Unadjusted	Adjusted^‡^	Unadjusted	Adjusted^‡^
Ever use of cigarettes	Yes	**1.37 (1.18–1.61)**	**1.32 (1.12–1.55)**	**1.08 (1.02–1.15)**	**1.08 (1.01–1.14)**	**1.18 (1.08–1.28)**	**1.28 (1.18–1.40)**
	No	1	1	1	1		
Ever use of e-cigarettes^†^	Yes	**1.31 (1.08–1.60)**	**1.23 (1.00–1.52)**	**1.09 (1.01–1.19)**	1.08 (1.00, 1.18)	**1.19 (1.06–1.33)**	**1.32 (1.18–1.49)**
	No	1	1	1	1		1
Ever use of HTP^†^	Yes	**1.85 (1.43–2.40)**	**1.78 (1.37–2.32)**	**1.24 (1.08–1.41)**	**1.21 (1.06–1.38)**	**1.43 (1.21–1.69)**	**1.58 (1.34–1.87)**
	No	1	1	1	1		1
Secondhand smoking at home	≥5 days/week	**1.63 (1.33–2.00)**	**1.63 (1.33–2.00)**	**1.14 (1.05–1.24)**	**1.13 (1.04–1.23)**	**1.50 (1.34–1.69)**	**1.42 (1.26–1.59)**
	1–4 days/week	1.09 (0.95–1.25)	1.08 (0.94–1.24)	**1.07 (1.01–1.12)**	**1.08 (1.02–1.14)**	**1.19 (1.09–1.30)**	**1.18 (1.08–1.29)**
	0 day/week	1	1	**1**	**1**	**1**	**1**
Secondhand smoking at school	≥5 days/week	**1.68 (1.37–2.06)**	**1.68 (1.37–2.07)**	**1.17 (1.06–1.31)**	**1.14 (1.03–1.27)**	**1.39 (1.22–1.59)**	**1.42 (1.24–1.62)**
	1–4 days/week	**1.40 (1.23–1.60)**	**1.42 (1.25–1.62)**	**1.21 (1.15–1.28)**	**1.19 (1.13–1.26)**	**1.14 (1.04–1.24)**	**1.13 (1.04–1.23)**
	0 day/week	1	1	**1**	**1**	**1**	**1**

^*^Significance at *p* < 0.05: Adjustment for multiple comparisons with Bonferroni correction.

^†^Electronic cigarettes: E-cigarettes, HTP: Heated Tobacco Product.

^‡^Adjusted for age, sex, obesity, residential area, family economic status, and physical activity. OR (95% CI): Odds ratio (95% Confidence interval).

According to Table 4, even after adjusting for all other variables, the use of all three tobacco products was associated with a higher risk of asthma, AR, and AD (AOR = 1.59, 95% CI: 1.17–2.15; AOR = 1.22, 95% CI: 1.05–1.42; and AOR = 1.64, 95% CI: 1.36–1.98) (Table 4). Sole use of HTP was strongly associated with asthma (AOR = 3.59, 95% CI: 1.47–8.78), and so was dual use of cigarette and HTP (AOR = 4.38, 95% CI: 2.44–7.87).

**Table 4:** Multiple logistic regression model of the association of tobacco use types with Asthma, Allergic Rhinitis, and Atopic Dermatitis.

Types of tobacco use	Asthma		Allergic Rhinitis		Atopic Dermatitis	
	OR (95% CI)		OR (95% CI)		OR (95% CI)	
	Unadjusted	Adjusted^‡^	Unadjusted	Adjusted^‡^	Unadjusted	Adjusted^‡^
**Ever use**						
Cigarettes only	**1.33 (1.11–1.59)**	**1.30 (1.08–1.56)**	1.02(0.94–1.10)	1.02(0.94–1.10)	**1.13 (1.00–1.27)**	**1.20 (1.07–1.35)**
E-cigarettes^†^ only	1.50 (0.91–2.46)	1.42 (0.86–2.34)	0.79 (0.63–0.99)	0.79 (0.63–1.00)	1.19 (0.89–1.60)	1.34 (1.00–1.80)
HTP^†^ only	**3.73 (1.54–9.05)**	**3.59 (1.47–8.78)**	1.47 (0.79–2.75)	1.43 (0.76–2.69)	1.11 (0.44–2.79)	1.20 (0.49–2.98)
Cigarettes + E-cigarettes	1.21 (0.90–1.62)	1.14 (0.84–1.54)	1.10 (1.00–1.22)	1.10 (0.99–1.21)	1.10 (0.94–1.29)	**1.24 (1.06–1.46)**
Cigarettes + HTP	**4.52 (2.52–8.12)**	**4.38 (2.44–7.87)**	1.31 (0.88–1.94)	1.27 (0.85–1.90)	**2.18 (1.33–3.59)**	**2.34 (1.41–3.89)**
E-cigarettes + HTP	1.18 (0.31–4.46)	1.15 (0.30–4.37)	0.91 (0.53–1.56)	0.89 (0.52–1.53)	0.91 (0.37–2.23)	1.07 (0.44–2.61)
Cigarettes + E-cigarettes + HTP	**1.63 (1.22–2.19)**	**1.59 (1.17–2.15)**	**1.24 (1.07–1.45)**	**1.22 (1.05–1.42)**	**1.43 (1.19–1.72)**	**1.64 (1.36–1.98)**
Never use	1	1	1	1	1	1

^*^Significance at *p* < 0.05: Adjustment for multiple comparisons with Bonferroni correction.

^†^Electronic cigarettes: E-cigarettes, HTP: Heated Tobacco Product

^‡^Adjusted for age, sex, obesity, residential area, family economic status, and physical activity. OR (95% CI): Odds ratio (95% Confidence interval).

Among the group of asthmatic participants, those diagnosed with asthma and AR accounted for 40.9%. Moreover, the three allergic diseases co-existed in 19.2% of asthmatic participants. However, 80.6% of AR participants were diagnosed solely with this disease.

In this study, an increased risk of multi-morbidity was attributable to the use of conventional cigarettes (AOR = 1.98, 95% CI: 1.45–2.70), e-cigarettes (AOR = 1.83, 95% CI: 1.25–2.68), and HTPs (AOR = 2.48, 95% CI: 1.48–4.13) (Table 5). The multinomial regression analysis indicated that adolescents using tobacco products were more likely to suffer from allergic multi-morbidity of asthma, AR, and AD altogether. In addition, the AOR for having only AD relative to the use of conventional cigarettes, e-cigarettes, and HTPs were 1.34 (95% CI: 1.20–1.50), 1.37 (95% CI: 1.17–1.61), and 1.80 (95% CI: 1.44–2.26).

**Table 5:** Multinomial logistic regression model of the association of tobacco use with multi-morbidity of Asthma, Allergic Rhinitis (AR), and Atopic Dermatitis (AD).

	Asthma only	AR only	AD only	Asthma + AR	Asthma + AD	AR + AD	Asthma + AR + AD
Allergic Participants, n (%)	515 (3.3)	9543 (65.1)	2348 (15.5)	573 (3.9)	82 (0.5)	1495 (10.0)	273 (1.8)
Tobacco use	**OR (95% CI)**^**‡**^						
Ever use of cigarettes	1.11 (0.86–1.42)	1.06 (1.00–1.13)	**1.34 (1.20–1.50)**	**1.28 (1.01–1.63)**	1.57 (0.86–2.84)	1.11 (0.95–1.29)	**1.98 (1.45–2.70)**
Ever use of e-cigarettes^†^	0.86 (0.59–1.25)	1.05 (0.97–1.15)	**1.37 (1.17–1.61)**	1.36 (0.99–1.87)	1.45 (0.69–3.04)	1.19 (0.96–1.47)	**1.83 (1.25–2.68)**
Ever use of HTP^†^	1.54 (0.95–2.49)	**1.22 (1.06–1.40)**	**1.80 (1.44–2.26)**	**1.70 (1.06–2.74)**	**3.87 (1.24–12.10)**	1.18 (0.83–1.68)	**2.48 (1.48–4.13)**

^*^Significance at *p* < 0.05: Adjustment for multiple comparisons with Bonferroni correction.

^†^Electronic cigarettes: E-cigarettes, HTP: Heated Tobacco Product.

^‡^Adjusted for age, sex, obesity, residential area, family economic status, and physical activity. OR (95% CI): Odds ratio (95% Confidence interval).

## Discussion

This study investigated the association between tobacco product use and allergic diseases in a school-based sample of adolescents in Korea.

In our study, active tobacco smoking in adolescents was associated with asthma. This result is in concordance with that of previous studies^7,24–26^. In a health study on 46,182 African American women, the incidence of asthma among current active smokers was higher than that among never smokers (HR: 1.43, 95% CI: 1.15–1.77)^24^. The effect of active cigarette smoking on adolescent-onset asthma symptoms was also shown in a German cohort study with the adjusted incidence risk ratio of 2.56 (95% CI: 1.55–4.21)^25^.

Our study pointed out the association between active smoking and AR. This result was consistent with that of some previous studies. For example, an epidemiological study^26^ of 14,578 French adolescents showed that those with active smoking were 2.95 times more likely to suffer from AR than the others (95% CI: 1.58–5.49). A recent meta-analysis of 97 studies on tobacco exposure and allergic conditions^27^ reported that active smoking among children and adolescents was significantly associated with the risk of AR.

Like our study, several studies demonstrated that smoking was associated with a higher risk for AD. Saulyte *et al*.^27^ examined 91 studies on allergic dermatitis and revealed that both active and passive smoking were associated with allergic dermatitis in children and adolescents. In a Korean study^28^, adolescents who smoked more than 20 days/month were more likely to develop AD than non-smokers (AOR = 1.18, 95% CI: 1.07–1.29).

The link between conventional cigarette smoking and allergic diseases has been well studied, whereas little research has been conducted on the association of e-cigarettes or HTPs with allergic diseases. A recent cross-sectional study^29^ of youths found that e-cigarette users developed more asthmatic symptoms than non-smokers. Furthermore, since HTPs have recently been introduced to the tobacco market, the (likely) mechanisms that link HTP use to the onset of allergic diseases have not been elucidated.

According to the recent data in the US, multiple tobacco product use was prevalent among adolescents. In 2018, 11.3% of high school students (41.7% of current users) reported using two or more tobacco products^30^. Our findings pointed out that triple use was most prevalent among HTP users (nearly 82.4%). According to a study of US youths, irritable or restless feelings due to tobacco withdrawal were 1.3 times more likely to be reported among multiple tobacco product users than among those using only one type of product^31^. This suggested that multiple tobacco users were possibly dependent of nicotine. Furthermore, their frequency of tobacco use increased, suggesting increased nicotine exposure and vulnerability to negative tobacco-related health effects^32^.

In this study, triple use of tobacco products was significantly associated with the risk of asthma, AR, and AD respectively. Multiple use may have a significant detrimental effect on health due to increased exposure to nicotine and/or toxicants. To further assess the risk for developing any allergic disease, it is necessary to understand the characteristics of multiple tobacco users and their use patterns.

Either comorbidity or multi-morbidity in patients was associated with an increase in physician/nurse appointments, visits to emergency department, or prescribed medication load^33^. Asthma, AR, and atopic disease are known to be inter-related, and multi-morbidity of allergic disease is commonly observed in adolescents^34–36^. Multi-morbidity can be observed in any allergic disease; however, this study shows an increased risk of reporting three allergic diseases concomitantly in smokers, compared to non-smokers.

Additionally, ever use of any tobacco product was significantly associated with a sole diagnosis of AD in our study participants. AD commonly manifested as a beginning of the ‘atopic march’; it is often followed by subsequent development of allergic rhino-conjunctivitis and asthma^37^. Therefore, it is plausible that local cutaneous sensitization may induce the systemic immune response with migration of sensitized T cells into the mucous membranes of the nose and lung through the circulatory system. This activates eosinophils and epithelial cells, IgE production, proliferation of mast cell, and smooth muscle observed in asthmatic patients^37,38^.

Tobacco use, in any form, may induce an inflammatory and immune response associated with increased exposure to allergens. Although several pathophysiological possibilities have been suggested, multi-morbidity of allergic disease in smokers has not been widely considered. Given the increasing prevalence and the importance of allergic diseases in adolescents, further monitoring and investigation on this mechanism is needed.

### Limitations

There are some limitations which should be considered. The cross-sectional design limited the possibility of establishing causal relationships between tobacco product use and allergic diseases, although this study had a large representative sample. A longitudinal research design would be more desirable to examine such causal relationships. Since our survey consisted of a large participants, objective indicators, such as serum IgE levels, eosinophil counts, or serum cotinine levels, could not be measured. Additionally, the questionnaire was not designed to investigate detailed features of allergic diseases (e.g. frequency, severity, or associated symptoms). Furthermore, adolescents tend to under-report their smoking behavior to conceal their delinquency, thus affecting the accuracy of measurement. Another limitation is that this study relied on diagnoses reported by patients rather than physician diagnoses. However, the generalizability of the study results is high because the study had a representative sample and design, as well as a high response rate of over 95%.

## Conclusion

In conclusion, our study findings indicated that the use of multiple tobacco products, including HTPs, might be a risk factor for asthma, allergic rhinitis, and atopic dermatitis. Additionally, the consistent use of each tobacco product we examined was linked to multi-morbidity of asthma, allergic rhinitis, and atopic dermatitis. An initial diagnosis of atopic dermatitis can lead to a subsequent systemic immune response. Healthcare professionals should pay close attention to smoking in adolescents because it is a potential contributor to the development of allergic diseases. To examine whether these associations are transient, further research with a longitudinal study design should be conducted.

## Materials and Methods

### Data and study participants

The present study used data from the Korea Youth Risk Behavior Survey of students aged 12–18 years from middle and high schools in Korea conducted in 2018. This government-approved survey^39^ has been annually performed since 2005. It is used by the Korea Centers for Disease Control and Prevention for monitoring adolescent health behaviors, including alcohol consumption, smoking, obesity, and physical activity. The survey was developed to produce a nationally representative sample of Korean adolescents with a stratified multi-stage cluster sampling design.

**Figure 1:**
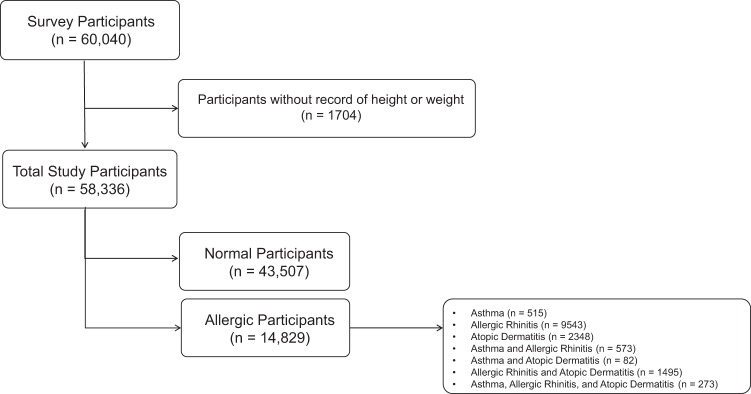
A schematic illustration of the participant selection process.

Initially, 400 middle schools and 400 high schools were randomly selected from 117 strata formed based on the administrative divisions and school types. Then, one class in each grade were randomly selected at each selected school. Out of a total of 2,850,118 students, a representative sample of 62,823 was selected from 5625 schools (middle and high) in Korea. Among the representative sample of 62,823 students recruited, 60,040 students agreed to participate in the survey, giving a high response rate of 95.6%. Besides, any student not providing information about their height or weight was excluded from the study. Therefore, the final study participants included 58,336 students (Fig. 1).

Students filled in online informed consent forms before participating in the survey in computer rooms. Parental consent was waived, as the survey was carried out by the Ministry of Education at schools with large numbers of students. The institutional Review Board of the Catholic University of Korea approved this study (as exempted from review, MC19ZESI0073).

### Measures

As the study outcomes, allergic diseases (asthma, AR, or AD) were identified based on the participant’s self-reported diagnoses. Participants were asked the following question: ‘Within the past 12 months, have you been diagnosed with asthma/allergic rhinitis/atopic dermatitis by a medical doctor?’(yes, no).

This study considered three tobacco products, namely conventional cigarettes, e-cigarettes, and HTPs. Students were determined to have a history of cigarette use if they answered ‘yes’ to the question ‘Have you ever smoked a cigarette in your lifetime, even one puff?’. To identify a history of e-cigarette or HTP use, we asked the students if they had ever used an e-cigarette or a heated tobacco product (e.g., IQOS, glo, or lil), excluding conventional cigarettes and e-cigarettes in their lives. Ever use of two or three of the tobacco products was defined as dual or triple use. Secondhand smoking at home was assessed by the following question: ‘Within the past seven days, how many days have you spent time with someone who smoked in your home (either family member or guest)?’ Meanwhile, secondhand smoking at school was investigated according to the question: ‘In the past seven days, have you ever been in close contact with an active smoker at school?’ Secondhand smoking at home and school was divided into three groups: 0 day/week; 1–4 days/week; and ≥5 days/week.

General characteristics, such as age, sex, obesity, residential area, family economic status, and physical activity, were also assessed and used as covariates in the logistic regression models. In terms of body mass index (kg/m^2^), the study participants were divided into four groups according to the Centers for Disease Control and Prevention guidelines for children and teenagers^40^. Four groups included obese (≥95^th^ percentile), overweight (≥85^th^ percentile and <95^th^ percentile), normal or healthy weight (≥5^th^ percentile and <85^th^ percentile), and underweight (<5^th^ percentile). The ‘residential area’ variable had three values, including large cities, medium or small cities, and provinces. Students were asked “What is the economic condition of your family?” in the questionnaire. Five levels of family economic status were specified in the questionnaire as high, mid-high, middle, mid-low, and low, and they were regrouped as high, middle, and low for the sake of analysis. Physical activity was evaluated as the number of days per week with ≥60 minutes of moderate-intensity activity that caused a slight increase in one’s breathing or pulse rate. Students were asked “During the last seven days, on how many days did you do more than 60 minutes of moderate-intensity physical activity per day?”. Responses regarding the frequency of physical activity ranged from none to seven days per week. Participants engaging in moderate-intensity activity at least five days per week were considered as physically active. Physical activity criteria were based on the moderate level physical activity guideline stated in the International Physical Activity Questionnaire.

### Statistical analyses

First, descriptive statistics of general characteristics among participants were presented as unweighted frequencies and weighted percentages. In addition, chi-square tests were used to examine the difference in the prevalence of each allergic disease by general characteristics, smoking-related characteristics, and tobacco use type. Subsequently, simple logistic regression analyses were conducted to calculate odds ratios (ORs) and adjusted ORs (aORs) for asthma, AR, and AD in the past 12-months based on the use of tobacco product; 95% confidence intervals (CI) were also calculated. Finally, multinomial logistic regression analyses were performed to identify the associations of tobacco product use with risk of allergic diseases. Bonferroni for post-hoc analysis was used for multiple comparisons. Adjusted models included age, sex, obesity, residential area, family economic status, and physical activity as covariates. Complex sampling modules were reflected and recommended weights were applied in this study. All statistical analyses were performed using SPSS version 26.0 (IBM, Armonk, NY, USA). The level of statistical significance was set at *p* < 0.05.

## Data Availability

The datasets analyzed during the current study are publicly available. Individual researchers need to gain access to download the dataset via the official website http://yhs.cdc.go.kr.
